# Distinctive factors contributing to psychological distress in second‐generation offspring of Holocaust survivors: Posttraumatic stress and sense of coherence

**DOI:** 10.1002/jts.23173

**Published:** 2025-06-12

**Authors:** Laura Nohr, Yuriy Nesterko, Freya Specht, Nadine Stammel, Ingrid Sotelo, Maria Böttche

**Affiliations:** ^1^ Department of Education and Psychology Division of Clinical Psychological Intervention Freie Universität Berlin Berlin Germany; ^2^ Center ÜBERLEBEN Berlin Germany

## Abstract

The psychological impact of historical trauma can be passed on to future generations. The simultaneous presence of historical and individual trauma may increase psychological distress, especially in older adults. Older age potentially represents a phase of life with increased challenges, distress, life review, and reminiscence. Though both historical and individual trauma appear to contribute to psychological distress, a strong sense of coherence (SOC) may reduce psychological distress and posttraumatic stress in older age and in the context of historical trauma. We conducted a cross‐sectional online survey among offspring of Holocaust survivors (OHS) from Germany, Israel, and the United States, focusing on the second generation and individuals aged 60–80 years who reported having survived individual trauma. Descriptive statistics, zero‐order correlation analyses, and multiple regression were used to investigate factors influencing psychological distress, including gender, age, posttraumatic stress disorder (PTSD) symptoms, past victimization, family Holocaust knowledge, and SOC (balance, manageability, and reflection). The sample comprised 116 participants (70.1% female‐ identified, *M*_age_ = 67.85 years, *SD* = 4.45, range: 60–79 years). Multiple regression indicated that PTSD symptoms, *B* = 2.78, β = .58 (*SE* = .37), *p* < .001, and manageability, *B* = −0.54, β = −.20 (*SE* = .25), *p* = .034, were significantly associated with psychological distress. The final model accounted for 50.5% of the total variance in current psychological distress among older second‐generation OHS. These findings highlight the importance of individual risk and protective factors in understanding distress among older people in the context of historical trauma.

As populations age worldwide, both individuals and societies face distinct challenges in supporting the health of older adults. Mental health is an important part of health and well‐being in all age groups (World Health Organization, 1989). In older age, people have accumulated different risk and protective factors that influence their mental health (Zaidi, 2014). Moreover, older individuals are frequently confronted with age‐specific life events and risk factors that can increase psychological distress, such as a general decline in overall health and autonomy or the loss of family members and friends (Taylor et al., 2018), resulting in a high prevalence of mental disorders in samples of older adults (Andreas et al., 2017). As psychological distress is a well‐known general risk factor for health problems, including problems related to mental health (Barry et al., 2020), it is especially important to understand the determinants of psychological distress in specific subgroups of older adults to address their specific needs effectively. Therefore, the current study aimed to investigate psychological distress in older offspring of Holocaust survivors (OHS) and to foster the understanding of the impact of potential risk and protective factors associated with historical and individual trauma.

## Psychological distress, trauma, and aging

Children of Holocaust survivors have now reached older adulthood and are facing unique challenges in navigating their identities and psychological well‐being (Shmotkin et al., 2011). Indeed, this population is confronted not only with their individual biography and the general process of aging but also with the impact of the collective trauma of their ancestors (Greenblatt‐Kimron et al., 2024), which may increase their psychological distress in older age. *Nonspecific psychological distress* refers to transdiagnostic and heterogeneous cognitive, behavioral, emotional, and psychophysiological symptoms (Kessler et al., 2002) that are important indicators of impaired well‐being and mental health. The enduring psychological impact of the Holocaust continues to shape the lives of survivors and their descendants (Kellermann, 2009), with this long‐lasting, transgenerational impact being described as *historical trauma*. The term historical trauma refers to the experience of massive collective trauma among an entire population (Brave Heart, 2003). Its designation as transgenerational implies that members of an affected community might experience trauma‐related symptoms or other clinical and nonclinical consequences (e.g., learned mistrust, historical thoughts of loss) without having experienced the specific traumatic event themselves (Mutuyimana & Maercker, 2023). Historical traumatization has also been conceptualized as a past‐oriented perception of existential threat reflecting the memory of collective trauma, which might impact current emotions, thoughts, and behavior (Hirschberger et al., 2016).

With regard to the historical trauma of the Holocaust, research has investigated the mental health of Holocaust survivors and their offspring. In the last several decades, meta‐analyses and reviews have reported conflicting findings regarding the general mental health of OHS. Though earlier meta‐analyses found that nonclinical OHS do not appear to have more mental health problems or indications of secondary traumatization than their peers with ancestors who were not Holocaust survivors (e.g., Sagi‐Schwartz et al., 2008), more recent literature has reported intergenerational consequences of the Holocaust, such as posttraumatic stress symptoms in the second‐generation OHS (Payne & Berle, 2021). To explain these inconsistent findings, some scholars have argued that there may be specific subgroups of OHS among whom specific parental and child characteristics and their interactions lead to a higher vulnerability to negative mental health outcomes (e.g., insecurity about one's competence; Danieli et al., 2017). This transmission might be based on interpersonal relationships; parenting and attachment; parental communication about the traumatic event; and other genetic, epigenetic, social, and cultural influences (Kellermann, 2009, as cited by Shrira et al., 2025). Further, differences between clinical and nonclinical samples of OHS have been discussed (e.g., Kellerman, 2001). These assumptions were summarized in the concept of *resilient*‐*vulnerability*, which describes how OHS show resilience and vulnerability in multiple facets and may view their vulnerabilities as both a weakness and a source of strength (Kidron et al., 2023); for example, OHS perceive the heightened response to recent civil unrest and protest as a strength to remain prepared and active (Shrira, 2015; Shrira et al., 2011).

As further mechanisms of transgenerational transmission, research has frequently examined secondary traumatization (i.e., symptoms of distress resulting from close contact with a traumatized individual) and parental posttraumatic stress disorder (PTSD). In a meta‐analysis, Lambert et al. (2014) found that parental PTSD was associated with offspring distress, though the study did not focus specifically on Holocaust survivors. Greenblatt‐Kimron et al. (2024) found higher rates of probable PTSD in OHS compared to peers without a familial Holocaust background. Furthermore, OHS have been found to show higher secondary traumatization than comparator groups (Hoffman & Shrira, 2019), especially when second‐generation Holocaust survivors perceived parental Holocaust‐related communication as intrusive (Shrira, 2016).

Studies with a more transdiagnostic focus have reported higher levels of psychological distress and a higher vulnerability to such distress among OHS compared to peers without a Holocaust background; for example, Shrira and Felsen (2021) reported this finding in the context of a life‐threatening event (i.e., the COVID‐19 pandemic). This difference has been especially pronounced when a given stressor or traumatic event mirrored Holocaust survivors’ sense of threat (e.g., the possibility of World War III or nuclear war; Greenblatt‐Kimron et al., 2023).

Regarding older adults and the process of aging, secondary traumatization shapes more negative perceptions of the aging process (Shrira, 2016). Furthermore, second‐generation Holocaust survivors with parental PTSD describe their aging process as less successful compared with peers without parental PTSD (Hoffman & Shrira, 2019). Regardless of the intergenerational transmission of parental traumatization, studies mostly point to a negative impact of trauma and PTSD on the aging process, the physical health and functional performance of older adults, dementia (Jakel, 2018), and premature mortality (Solomon et al., 2014). In addition, OHS report a heightened preoccupation with potential surrounding threats, referring both to current threats and to historical traumatization related to the Holocaust (Greenblatt‐Kimron et al., 2023). Wohl and Van Bavel (2011) showed that the association between Jewish group identification and PTSD symptoms was mediated by family discussions of victimization. Moreover, OHS have been found to hold a more negative view of the world and to show an increased preoccupation with various current surrounding threats (e.g., the Iranian nuclear threat; Shrira, 2015).

Consequently, PTSD, individual trauma, and historical trauma might act as additional stressors that reinforce existing psychological distress and other risk factors. The interactions among individual trauma, historical trauma, and secondary traumatization have been described within the framework of the *polyvictimization theory*, according to which individuals who experience multiple types of trauma are more likely to show psychological problems than those who experience fewer types (Hamby et al., 2020).

## Protective factors and sense of coherence

Studies investigating the psychological impact of historical trauma have focused not only on negative consequences but also on salutogenesis (i.e., the human capability to preserve or recover health after critical life events or other severe life stressors; Bachem & Maercker, 2016). OHS have been found to show a stronger sense of optimism and hope, higher life satisfaction and quality of life, and resilience compared to peers without a Holocaust background (Shrira, 2015; Shrira et al., 2011) and to maintain resilience in middle age (Shrira, 2016). In the context of adversities, theories on salutogenesis have been expanded by the concept of *sense of coherence* (SOC), defined as the ability to use internal and external resources to overcome adversity (Antonovsky, 1987, 1996). SOC facilitates one's view of the world as comprehensible, manageable, and meaningful (Antonovsky, 1987) and fosters resilience to stressors (Galletta et al., 2019). In the context of historical trauma, a higher SOC was shown to predict fewer history‐related symptoms in American Indian youth (Evans & Davis, 2018). In a sample of adult offspring of Lithuanian survivors of political violence, SOC and posttraumatic stress reactions were significantly negatively correlated (Kazlauskas et al., 2017). Studies have shown that SOC increases with age, with older adults showing higher SOC scores than younger age groups (Nilsson et al., 2010). In older Holocaust child survivors, SOC was found to moderate the association between traumatic experiences during the war and posttraumatic stress (van der Hal‐van Raalte et al., 2008). In sum, previous literature suggests a positive influence of SOC on mental health in the context of historical trauma and evidence of higher SOC levels in older age.

## Study aims and hypotheses

In the current study, we examined a sample of older second‐generation Holocaust survivors who reported having experienced individual trauma during their lifetime. We expected to find that aspects of historical trauma (e.g., past victimization, knowledge about family members’ Holocaust experience), as well as posttraumatic stress symptoms stemming from individual trauma, would demonstrate positive associations with levels of psychological distress in older OHS. Moreover, we examined SOC as a factor that might increase resilience and demonstrate a negative association with psychological distress in older age.

## METHOD

### Participants and procedure

We conducted an online cross‐sectional survey, using Unipark software (Questback GmbH, 2021), from November 2021 to April 2022 to recruit a community sample of second‐ and third‐generation OHS. Using convenience sampling, the survey was distributed via a link posted on social media (e.g., postings in relevant Facebook groups); mailing lists and newsletters from Jewish communities and local synagogues in Germany, Israel, and the United States; local welfare organizations; local nongovernmental organizations (NGOs); and personal contacts in the three countries listed. The survey was offered in three languages (English, German, and Hebrew), and participants could choose which language to use according to their preferences. After opening the survey link, potential participants were informed about the aims and duration of the study; storage, use, and publication of the data; the voluntary nature of their participation; and the right to cease participation without giving reasons and with no negative consequences. After actively giving informed consent, participants completed the online survey, with an average completion time of 14 min. First, participants were asked for sociodemographic information and details about their identity as an OHS. Automatic filters verified the inclusion and exclusion criteria, and only participants who fulfilled all study criteria were directed to the online survey. No financial compensation or other benefits were provided, and there was no risk or negative consequence involved in participation or nonparticipation. All questions related to sociodemographic variables and measures were available in English, German, and Hebrew. When translations were unavailable, two professional translators independently translated and back‐translated all material to ensure accuracy and consistency. The two translated versions were compared, and any differences were discussed until a consensus was reached. The study procedure was approved by the ethics committee of the Department of Education and Psychology at Freie Universität Berlin, Germany (040/2021).

To be eligible for this secondary analysis of a larger dataset that included 496 second‐ and third‐generation OHS, participants had to be 60–80 years old; identify as the child of a Holocaust survivor; and have grown up in Germany, Israel, or the United States. We defined older adults as people aged 60 years or older according to a United Nations (U.N.) definition (U.N. Department of Economic and Social Affairs–Population Division, 2020). Participants who reported being the offspring of at least one parent who had survived the Holocaust were included in the current analysis, regardless of whether one or more of their grandparents were also Holocaust survivors. Participants were asked individually if their mother, father, and maternal and paternal grandparents had survived the Holocaust. This resulted in a range of one to six family members who survived the Holocaust, with an average of 2.59 (*SD* = 1.41) family members in the current subsample. Additionally, only participants who stated that they had experienced at least one lifetime personal traumatic event were included.

The final convenience sample consisted of 116 second‐generation Holocaust survivors from Jewish communities, with a mean participant age of 67.85 years. Most of these participants were female (70.1%), highly educated (66.1% with a university degree), married (68.6%), and had grown up in Israel (70.1%). The majority of participants were living in Israel (65.3%) when they completed the survey. Although we did not focus on a clinical sample, 20.3% of participants screened positive for PTSD, and the mean of reported PTSD symptoms was 1.90 (*SD* = 1.64). On average, participants reported general distress scores below the cutoff for moderate distress measured with the Kessler Psychological Distress Scale (K10; Kessler et al., 2002), and high levels of knowledge about family members’ Holocaust experiences (i.e., “How much do you know about your family members’ Holocaust experiences?”); past victimization, measured using the Past Victimization subscale of the Multidimensional Existential Threat Scale (MET; Hirschberger et al., 2016); and SOC, using with the Sense of Coherence Scale–Revised (SOC‐R; Bachem & Maercker, 2016). Descriptive results for the main variables are presented in Tables 1 and 2.

**TABLE 1:** Sociodemographic characteristics of the sample

Variable	*n*	%
Gender		
Female	82	70.7
Male	34	29.3
Educational attainment		
Junior high school/middle school	1	0.9
Secondary school/high school	8	6.9
College	27	23.3
University	76	65.5
Vocational school	4	3.4
Marital status		
Single	8	6.9
Partnership	7	6.0
Married	79	68.1
Widowed	6	5.2
Divorced	15	12.9
Other	1	0.9
Country of origin		
Germany	10	8.6
Israel	82	70.7
United States	24	20.7
Current country of residence		
Germany	14	12.1
Israel	77	66.4
United States	24	20.7
Other	1	0.9
Probable PTSD		
No	73	79.7
Yes	24	20.3
Number of familial Holocaust survivors		
1	22	19.0
2	50	43.1
3	20	17.2
4	10	8.6
5	5	4.3
6	9	7.8

*Note*: *N* = 116. PTSD = posttraumatic stress disorder.

**TABLE 2:** Descriptive characteristics of the sample and zero‐order correlations

Variable	1	2	3	4	5	6	6a	6b	6c	Range	*M*	*SD*
1. Age (years)	–	−.104	−.008	−.133	−.051	.033	−.002	.081	.000	60–79	67.85	4.45
2. Stress (K10)		–	.683^***^	.070	.249^*^	−.120^*^	−.086	−.439^***^	−.138	10–42	19.59	7.94
3. PTSD symptoms (PC‐PTSD‐5)			–	.170	.197^*^	−.225^*^	−.015	−.433^***^	−.087	0–5	1.90	1.64
4. Knowledge about Holocaust experiences				–	.157	.021	−.029	−.017	.106	2–10	7.24	2.05
5. Past victimization (MET)					–	.119	.192^*^	−.049	.123	5–20	15.46	3.77
6. Sense of coherence (SOC‐R total)						–	.738^***^	.796^***^	.793^***^	24–64	47.18	7.01
6a. SOC‐R Balance							–	.315^***^	.333^***^	6–20	13.13	3.31
6b. SOC‐R Manageability								–	.564^***^	10–25	18.31	2.96
6c. SOC‐R Reflection									–	5–20	15.74	2.79

*Note*: *N* = 116. PTSD = posttraumatic stress disorder; PC‐PTSD‐5 = Primary Care PTSD Screen for *DSM‐5*; K10 = Kessler Psychological Distress Scale; MET = Multidimensional Existential Threat Scale; SOC‐R = Sense of Coherence Scale–Revised.

* *p* < .05. ** *p* < .01. *** *p* < .001.

### Measures

#### Sociodemographic information

Sociodemographic variables for the current study included age, gender, country of origin, current country of living, educational attainment, marital status, identification as an OHS, and number of familial Holocaust survivors. In addition, one item asked participants about their knowledge regarding family members’ Holocaust experiences (i.e., “How much do you know about your family members’ Holocaust experiences?”), with responses rated on a scale of 1 (*nothing at all*) to 10 (*very much*).

#### Psychological distress

Global psychological distress was assessed using the self‐report 10‐item Kessler Psychological Distress Scale (K10; Kessler et al., 2002), a widely used, reliable, and valid screening tool (Kessler et al., 2003). The one‐dimensional scale was constructed to detect psychological distress in the upper range of the general population and has demonstrated high discriminatory validity to detect anxiety disorders and depression and excellent internal consistency (Cronbach's α = .92; Kessler et al., 2002, 2003). Items are rated on a 5‐point Likert scale ranging from 1 (*none of the time*) to 5 (*all of the time*), with higher scores indicating higher levels of psychological distress (range: 10–50). A score above 24 has been established to indicate moderate psychological distress. English, German, and Hebrew versions of the K10 were readily available. The German version has demonstrated high internal consistency (Cronbach's α = .90), good convergent validity with other psychopathological constructs like anxiety or a global severity index, and good sensitivity and specificity (i.e., 80% and 81%, respectively; Giesinger et al., 2008). The Hebrew version was provided by the Israeli Ministry of Health (Kessler et al., 2003). In the present study, internal consistency was very good, Cronbach's α = .93.

#### PTSD symptoms

The Primary Care PTSD Screen for *DSM‐5* (PC‐PTSD‐5; Prins et al., 2016) was used to assess PTSD symptoms and screen for probable PTSD. This self‐report measure is tied to symptom criteria listed in the *Diagnostic and Statistical Manual of Mental Disorders* (5th ed.; *DSM‐5*; American Psychiatric Association, 2013) and consists of two parts. First, respondents were asked whether they had ever been exposed to a potentially traumatic event (PTE); in this study, this question was used as a “filter” question for study eligibility. After defining PTE exposure and providing a list of examples, participants were asked, “Have you ever experienced this kind of event?”, with responses of “yes” coded as 1 and “no” coded as 0. Individuals who denied this item were not included in the current analysis. Then, people who reported having experienced at least one potentially traumatic event (PTE) were asked five additional dichotomous (i.e., “yes” or “no”) items, with each diagnostic criterion for PTSD represented by one item. The PC‐PTSD‐5 is a reliable and valid measure of probable PTSD that has shown high correlations with gold‐standard diagnostic interviews, as well as high acceptability, diagnostic accuracy, sensitivity, and specificity in male and female U.S. veterans (Bovin et al., 2021; Prins et al., 2016). Participants with a total sum score of 3 or higher are classified as having probable PTSD, with a maximized sensitivity of 100.0%, acceptable specificity (85.2%), and maximized efficiency (κ[0.5]) of .77 (Williamson et al., 2022). For regression analysis, we used the sum score of PTSD symptoms as a metric indicator of symptom severity and load regardless of whether a participant fulfilled the criteria for probable PTSD. English (Prins et al., 2016) and German (Cronbach's α = .73; Horstmann et al., 2024; Schäfer & Schulze, 2010) versions of the scale were readily available; a Hebrew version was translated using previously described procedures. In the present study, internal consistency was acceptable, Cronbach's α = .73.

#### Past victimization

The Multidimensional Existential Threat Scale (MET; Hirschberger et al., 2016) is a self‐report questionnaire that is used to measure existential threat as a multidimensional construct. The full version consists of 20 items across four subscales measuring individual, social, and political aspects of existential threat. In the present study, only the Past Victimization subscale was used to assess the memory of collective trauma (e.g., “I often think of the grievances caused to my people in the past”). This subscale consists of four items that were rated on a scale of 1 (*strongly disagree*) to 5 (*strongly agree*), which is different from the original scale scoring. One item was reverse‐coded, and higher scores were reflective of higher levels of past victimization. The MET has shown at least acceptable psychometric properties for all subscales (Cronbach's αs = .71–.83). The Past Victimization subscale has demonstrated good internal consistency (Cronbach's α = .82). Convergent and discriminant validity of the Past Victimization subscale have been shown using correlations with relevant constructs like in‐group attachment and religiosity, as well as patterns of correlations with different political ideologies, such as in‐group identification and support for peace (Hirschberger et al., 2016). English and Hebrew versions of the scale were readily available; a German version was translated using previously described procedures. In the present study, internal consistency for the MET Past Victimization subscale was high, Cronbach's α = .80.

#### SOC

SOC was assessed using the self‐report Sense of Coherence Scale–Revised (SOC‐R; Bachem & Maercker, 2016), which measures the individual perception and integration of positive and negative experiences to sustain and enhance health. The scale comprises 13 items across three subscales: Balance, which describes the ability to balance positive and negative experiences and feelings; Manageability, defined as the ability to adjust to difficult situations; and Reflection, referring to the ability to perceive connections by taking different perspectives. Items are rated on a 5‐point Likert scale ranging from 1 (*not at all true*) to 5 (*extremely true*). The SOC‐R has been found to be a valid and reliable instrument and demonstrated acceptable psychometric properties in a sample of older adults in Switzerland (McGee et al., 2018). A confirmatory factor analysis (CFA) confirmed the proposed three‐factor structure with a second‐order factor. In the Swiss sample, the SOC‐R demonstrated low‐to‐good internal consistency (Cronbach's αs = .54–.78). Other positive and negative psychological concepts were used to demonstrate convergent and discriminant validity (*r*s = .31–.49, *p*s < .01), and, as theoretically assumed, the SOC‐R moderated the association between early‐life adversities and mental health in the Swiss sample. English and German versions of the SOC‐R, which have previously demonstrated overall satisfactory internal consistencies in two different samples (Cronbach's αs = .63–.81; Bachem & Maercker, 2016), were readily available; the measure was translated into Hebrew using previously described procedures. In the current study, internal consistency was acceptable for the total score, Cronbach's α = .79, and ranged from low to acceptable for the Balance, Cronbach's α = .64; Manageability, Cronbach's α = .57; and Reflection, Cronbach's α = .83, subscales.

### Data analysis

An a priori power analysis using G*Power (Version 3.1.9.7; Faul et al., 2009) yielded a necessary sample size of 109 to detect small effects. Descriptive statistics for the sample were calculated. Zero‐order correlation analyses were used to explore the data structure and the associations between the variables of interest. To test our hypotheses, we conducted a multiple regression analysis. The variance inflation factor (VIF) was used to detect multicollinearity between the predictors. The data did not contain any missing values. All analyses were performed using R (Version 4.1.3) and RStudio (Version 2024.04.2 Build 764).

## RESULTS

Descriptive results are presented in Table 1. Most zero‐order correlations between the variables of interest were in the expected directions (see Table 2). Age and knowledge about family members’ Holocaust experiences were not significantly correlated with any other variables of interest. The VIF for the multiple regression (see Table 3) ranged from 1.035 for age to 2.015 for SOC‐R Manageability, indicating small intercorrelations between the predictors and no relevant multicollinearity.

**TABLE 3:** Multiple regression to predict psychological distress of second‐generation Holocaust survivors in older age

Variable	*B*	*SE*	95% CI	β	*p*
(Intercept)	30.054	9.322	[11.574, 48.534]	.166	.002
Age	−0.145	0.119	[−0.381, 0.091]	−.081	.226
Gender^a^	−1.020	1.213	[−3.424, 1.384]	−.059	.402
PTSD symptoms	2.782	0.371	[2.046, 3.517]	.576	<.001
Knowledge about family members’ Holocaust experience	−0.236	0.268	[−0.767, 0.295]	−.061	.380
Past victimization	0.212	0.146	[−0.078, 0.502]	.101	.151
SOC‐R Balance	0.335	0.175	[−0.013, 0.682]	.139	.059
SOC‐R Manageability	−0.536	0.250	[−1.031, −0.041]	−.200	.034
SOC‐R Reflection	−0.046	0.240	[−0.521, 0.430]	−.016	.849

*Note*: *N* = 116. Model statistics: *R* ^2^ = .539, *R* ^2^_adj_ = .505, *F*(8, 107) = 15.63, *p* < .001. CI = confidence interval; PTSD = posttraumatic stress disorder; *SE* = standard error; SOC‐R = Sense of Coherence Scale–Revised.

^a^ 1 = female, 2 = male.

* p < .05. ** *p* < .01. *** *p* < .001.

Self‐reported PTSD symptoms were significantly associated with psychological distress among older second‐generation OHS, *B* = 2.78 (*SE* = 0.37), β = .58 *p* < .001. The manageability facet of SOC was also significantly associated with psychological distress, *B* = −0.54 (*SE* = 0.25), β = −.20 *p* = .034. Age, gender, past victimization, knowledge about family members’ Holocaust experiences, SOC‐R Balance, and SOC‐R Reflection were not significantly associated with psychological distress. The final model accounted for 50.5% of the total variance in current psychological distress among older second‐generation OHS and was statistically significant, *R* ^2^_adj_ = .505, *F*(8, 107) = 15.63, *p* < .001.

## DISCUSSION

Societies worldwide are currently in a state of demographic change, with relatively more adults of older age. To enable older adults to be fulfilled in their later years, it is crucial to understand the specific needs of subgroups of older adults and tailor prevention and intervention strategies accordingly. In the current study, we were interested in nonspecific psychological distress as an indicator of impaired well‐being and mental health in second‐generation OHS in older adulthood. This group is characterized by the historical trauma of the Holocaust their parents survived and its consequences for the Jewish identity. In addition, we focused on individuals who reported having experienced an individual traumatic event, some of whom screened positive for probable PTSD. Accordingly, our findings stem from a specific subgroup of older adults that are characterized by a trauma load that includes historical and individual trauma but not a clinical sample per se. The mean score for psychological distress was below the cutoff for moderate psychological distress. Still, over 20% of the sample screened positive for probable PTSD, which is higher than the prevalence among older adults not affected by historical trauma (Greenblatt‐Kimron et al., 2024; Pless Kaiser et al., 2019).

In our analysis, self‐reported PTSD symptoms exerted a strong negative influence on psychological distress. This seems reasonable based on the conceptual overlap between PTSD symptoms and symptoms of psychological distress. Further, this is in line with the polyvictimization theory (Hamby et al., 2020) and highlights the direct impact of PTSD symptom load on distress and well‐being in older age in the context of historical and individual trauma. Thus, posttrauma prevention and early intervention efforts might reduce the negative impact of trauma on individual biographies and mental health in older age, especially in populations affected by historical trauma and polyvictimization. Equally, older adults affected by PTSD should be offered trauma‐focused psychotherapy as the gold‐standard treatment for PTSD, with individual age‐related modifications if necessary (Phoenix Australia–Centre for Posttraumatic Mental Health, 2020).

Variables representing aspects of historical trauma were not significantly associated with psychological distress. This finding is not in line with previous research in other groups affected by historical trauma (e.g., Skrodzka et al., 2021). In the current study, the way in which older adults reflected upon Jewish life and their parents’ past traumatic experiences did not add to their current psychological distress beyond the impact of PTSD symptom load. PTSD symptoms and past victimization were correlated with each other (*r* = .197), which hints at some shared variance between these concepts and might inform future investigations. In addition, knowledge about Holocaust experiences of family members did not add to psychological distress beyond the impact of PTSD symptom load in this sample of older OHS. Previous studies have shown the importance of secondary trauma and the way trauma is communicated within families as potentially important mechanisms of transmission to next generations. Recent studies have also investigated Holocaust centrality and its associations with mental health outcomes in OHS (Greenblatt‐Kimron et al., 2024). Centrality of events describes the extent to which a traumatic event becomes a reference point for everyday experiences (Berntsen & Rubin, 2006). Holocaust centrality was also discussed as a potential mechanism of transgenerational transmission of trauma in families of Holocaust survivors (Greenblatt‐Kimron et al., 2024). This concept might add to the field's understanding of psychological distress in older OHS and other groups affected by historical trauma. In general, the current study did not focus on the interaction between historical and individual trauma and how this interaction might affect mental health in older adults. Further, we did not investigate possible mechanisms of the transmission of historical trauma, such as secondary trauma or parental PTSD. Future studies should further investigate the associations among and mechanisms of aspects of historical trauma, individual trauma, PTSD symptom load, and general distress and well‐being in older adults.

Regarding potential resilience among older OHS, the construct of SOC, particularly its manageability facet, needs to be considered. Manageability represents an adaptive skill and was significantly associated with psychological distress. It appeared to show a protective influence, which is in line with previous cross‐sectional studies in other trauma‐affected populations (e.g., Behnke et al., 2019). Interestingly, the more cognitive facets of SOC assessed (i.e., balance and reflection) showed no influence on psychological distress in older second‐generation OHS. The ability to adjust to difficult situations and circumstances might be the more important resource in older age and may help individuals cope with both past and current challenges and support mental health and well‐being (Bachem & Maercker, 2016). This knowledge could shape effective and tailored interventions in the future. For instance, the positive impact of manageability might be enhanced by strengthening problem‐solving skills and self‐efficacy in older adults (Remm et al., 2023).

Participants’ sociodemographic characteristics (binary gender and age) were not significantly associated with psychological distress. Although female gender is a well‐known predictor of PTSD symptoms (Tortella‐Feliu et al., 2019), it was not significantly associated with psychological distress over and above the impact of self‐reported PTSD symptoms and manageability. Previous findings regarding age as a predictor of psychological distress and PTSD are more mixed (Creamer & Parslow, 2008; Jorm et al., 2005). In the present study, restricting participant age to 60–79 years might have influenced our findings by reducing the variance in this variable. Nevertheless, as we were interested in the mental health of older adults, it stands to reason that we did not find a significant association between age and psychological distress in the current sample.

In sum, the regression model explained 50.5% of the variance in psychological distress in older OHS, although only two of the predictors were significantly associated with the outcome. Adding more relevant concepts of historical trauma, like Holocaust centrality, present sense of threat, or secondary traumatization, might explain even more variance. Still, the considerable impact of PTSD symptoms on general psychological distress as an indicator and risk factor of mental health highlights the importance of prevention and PTSD‐specific interventions in a population characterized by polyvictimization. Adaptive skills like manageability might be one helpful strategy to cope with psychological distress in older age.

Some limitations need to be considered when interpreting the current findings. Due to the cross‐sectional design of the study, we cannot draw any conclusions about the directions of effects or causality. Moreover, the current sample was a convenience sample rather than a representative sample of second‐generation OHS in Germany, Israel, and the United States. People with specific characteristics might be attracted by an online survey on these topics. The majority of participants were female, highly educated, and from Israel. The recruitment strategy, which included advertising in Facebook groups, NGOs, synagogues, and similar avenues, may not have reached certain subgroups of individuals, such as those with high levels of stress, low levels of digital literacy, or connections to the community. As such, it is difficult to specify the bias our sampling method may have conferred, but we cannot generalize our findings to the entire cohort of older second‐generation OHS. In addition, we only used a screening instrument to assess PTSD symptoms and did not further explore individual trauma and its consequences related to participants’ mental health and personal history. Therefore, we can only report on PTSD symptoms and probable PTSD and cannot determine which individual traumatic events participants experienced and at what point in life these events occurred. Nevertheless, given the strong association between PTSD symptoms and psychological distress in our sample, we can assume a relevant association between the concepts.

Our study provides relevant information about the consequences of historical and personal trauma and psychological distress, as well as potential protective factors, such as manageability, in a nonclinical sample of older second‐generation OHS. Knowledge about the importance of PTSD symptoms and manageability in this population can inform research and care in older age and in distinct populations affected by historical trauma. In this regard, a particular focus on older adults is important because aging may challenge previous coping mechanisms, potentially exacerbate existing PTSD symptoms (Phoenix Australia–Centre for Posttraumatic Mental Health, 2020), and represent a time of life review informed by individual and historical trauma (see Zimmermann & Forstmeier, 2020).

To summarize, the present findings should be interpreted within their specific context and, at the same time, be tested in the future to explore their generalizability to other populations living with historical trauma. Additionally, the data of this study were collected before the October 7th, 2023, attacks in Israel. The newly experienced collective trauma caused by these attacks shows selectively negative effects on OHS and a stronger increase in probable PTSD in OHS compared to peers without direct exposure to the Holocaust (Shrira et al., 2025). Therefore, future studies might result in even more pronounced findings on PTSD and distress in older OHS. In conclusion, the present study adds to the knowledge regarding mental health and aging in a sample with specific characteristics of both historical and individual trauma. Future research should include longitudinal studies to gain a clearer understanding of the directions of the effects. In addition, more relevant concepts in the context of historical trauma should be added. Based on the current study, PTSD treatment in older OHS should be fostered, and manageability as an adaptive skill should be considered in future research and care.

## AUTHOR NOTE

This study report is based on a larger study supported by a grant from AMCHA Germany e.V.

The authors wish to thank Ruth Wandrei and Tabea Schumacher for their contributions to the data collection for the study. The authors would also like to thank Jowan Rashed for his support in language editing and manuscript feedback.

## OPEN PRACTICES STATEMENT

The study reported in this article was not formally preregistered. Neither the data nor the materials have been made available in a permanent third‐party archive; requests for the data or materials should be sent by email to the corresponding author at maria.boettche@fu‐berlin.de.
